# Editorial: Fungal Systematics and Biogeography

**DOI:** 10.3389/fmicb.2021.827725

**Published:** 2022-01-25

**Authors:** Dhanushka N. Wanasinghe, Peter E. Mortimer, Jadson D. P. Bezerra

**Affiliations:** ^1^Center for Mountain Futures, Kunming Institute of Botany, Chinese Academy of Sciences, Kunming, China; ^2^Department of Economic Plants and Biotechnology, Yunnan Key Laboratory for Wild Plant Resources, Kunming Institute of Botany, Chinese Academy of Sciences, Kunming, China; ^3^Setor de Micologia, Departamento de Biociências e Tecnologia, Instituto de Patologia Tropical e Saúde Pública (IPTSP), Universidade Federal de Goiás (UFG), Goiânia, Brazil

**Keywords:** taxonomic diversity, biogeographic distributions, molecular phylogeny, ecological roles, host-specificity

According to best estimates, it is predicted that ~2.2–3.8 million or more fungal species exist on Earth (Hawksworth and Lücking, 2017). Nevertheless, we know of only 146,000 (Bánki et al., 2021), suggesting that 96% of fungal species remain unknown. Owing to their abundance across all ecosystems, fungal taxa simply cannot be overlooked in any region (Wanasinghe et al., 2020). Most species of plant-associated fungi can be pathogens, endophytes, saprobes or epiphytes across a wide range of hosts in terrestrial as well as aquatic habitats (Põlme et al., 2020). Given the ubiquitous nature of fungi, additional taxonomic and ecological knowledge are prerequisites to understanding fungal biology and their environmental significance.

Fungi are experiencing considerable conversion pressures due to a variety of devastating activities, including the intensification of land use alongside growing human populations, environmental exploitation and pollution, invasive species and climate change challenges. Recent studies have shown that fungi are likely to be sensitive to environmental changes and global warming (Tedersoo et al., 2014; Casadevall et al., 2019; Nnadi and Carter, 2021). This may be triggering the extinction of a large number of species that cannot adapt fast enough to the rate of environmental change, imbuing the research community with a sense of urgency that future opportunities for study may not exist. If we are willing to confront these challenges and work to mitigate species loss, then a robust, updated fungal classification that enables clear taxonomic communication using extensive fungal sampling across different geographic regions will be needed (Hyde et al., 2020).

Past studies have shown that certain fungi form close relationships with plants through mutualistic or antagonistic interactions (van der Heijden et al., 2008). Based on these plant-fungi interactions, it is clear that fungi play a key role in shaping plant community compositions and distribution patterns along natural gradients. Numerous studies have attempted to document the biogeography of fungi at granular and regional scales. The findings of these studies indicate a high diversity of fungi in tropical regions, specifically in tropical forests, with a decline in diversity toward the northern latitudes (Tedersoo et al., 2014). Additional studies in fungal biogeography, adopting either regional or fine scale approaches as well as studies focusing on specific fungal groups, will further enrich our knowledge of the distribution of fungi. In support of this endeavor, we propose the Research Topic “*Fungal Systematics and Biogeography*” for both Reviews and Original Research manuscripts. In this Research Topic, we accepted 26 articles from authors on five continents with emphasis on Asian countries, based on taxonomic diversity, molecular phylogeny, ecological roles, biogeographic distributions, host-specificity and co-evolutionary relationships of fungi.

Micro fungi are less studied when compared to macro fungi, especially in poorly sampled regions like Yunnan Province, China. Mortimer et al. collected and investigated several micro-fungi in Yunnan province from dead woody twigs based on morpho-molecular approaches. The study of Mortimer et al. resulted in one new genus and four new species within Dothideomycetes (Ascomycota). Jiang et al. investigated bambusicolous fungi in southwest China and Thailand. *Immotthia* was for the first time established within Dictyosporiaceae (Dothideomycetes, Ascomycota) based on morphological and phylogenetic analyses, with a new *Immotthia* species. In addition, a new monotypic genus *Pseudocyclothyriella* was established to accommodate *Pseudocyclothyriella clematidis*, which is the latest synonym of *Pseudocoleophoma clematidis*. Both genera were investigated by detailed morphological characterizations of type materials and multi-gene phylogenetic analyses. Doilom et al. collected and recorded several Leptosphaeriaceae (Dothideomycetes, Ascomycota) species from Asteraceae in China. A new genus *Praeclarispora* and a new species *Sphaerellopsis artemisiae* sp. nov. were introduced within Leptosphaeriaceae based on ascospores morphology and multi-locus phylogenetic analysis. Studying species in Thelebolaceae (Leotiomycetes, Acomycota) is important because they can produce antifreeze proteins, ice-binding proteins and other secondary metabolites that are valuable for biotechnological exploitation. During an extensive diversity survey of microfungi in China, Zhang et al. introduced one new genus (Solomyces) and five new species belonging to the order Thelebolales.

*Arthrinium* (Sordariomycetes, Ascomycota) is a cosmopolitan genus characterized by basauxic conidiogenesis and apiospores on its asexual and sexual morphs, respectively. Senanayake et al. collected *Arthrinium*-like taxa from bamboo plants in Guangdong Province, China. Based on morphological characteristics and phylogenetic data, they introduced two new species and reported two new locality records of *Arthrinium*. Feng Y. et al. introduced four new species and seven existing species of *Arthrinium* obtained from 17 isolates from China. Among the known species, three sexual morphs and one asexual morph were recorded for the first time. All species were subjected to detailed morphological and phylogenetic analyses. Entomopathogenic fungi are ubiquitous in tropical rainforests and feature a high level of diversity. Wei et al. collected entomopathogenic fungi from forests in the Greater Mekong Subregion and introduced three new species to *Paraisaria* (Sordariomycetes, Ascomycota). Gao et al. identified and described a novel genus, *Parametarhizium* (Sordariomycetes, Ascomycota) with two new entomopathogenic species based on morphology and a multi-gene phylogenetic reconstruction. In addition, their potential as biocontrol agents was evaluated by applying a spore-based anti-insect assay against *Monolepta hieroglyphica* (Coleoptera). Magnaporthales (Sordariomycetes, Ascomycota) members can be pathogenic, saprobic or endophytic, mainly on monocotyledonous herbaceous plants. Their biogeography has received less attention. Feng J.-W. et al. described the biodiversity of endophytic Magnaporthales fungi from Poaceae across three latitudes in China and conducted a meta-analysis of the geography and ecology of Magnaporthales worldwide.

Wood-inhabiting fungi play an important role in wood degradation and nutrient recycling. During a survey of wood-inhabiting fungi in southern China, Zhao and Zhao introduced three species of *Trechispora* (Bartheletiomycetes, Basidiomycota) from rotten wood. Wood-inhabiting poroid and corticioid species are ecologically and economically important as decomposers, pathogens, and sources of food and medicines. Gafforov et al. provided the first comprehensive, thoroughly annotated checklist of species diversity of wood-inhabiting poroid and corticioid fungi in Uzbekistan. They compiled 790 fungal records collected from 1950 to 2020. Fungi play central roles in the decomposition of lignin and other recalcitrant compounds. Osono et al. investigated the diversity and geographic pattern of ligninolytic fungi on *Castanopsis sieboldii* (Fagaceae) in Japan. They showed that the ligninolytic activity of fungi on leaf litter was greater at sites with higher mean annual temperature and precipitation where the latitudes and longitudes are lower. Osono et al. suggest that not only the geographic location and climatic conditions but also the biogeographic patterns of distribution of ligninolytic fungi influence the decomposition of lignin in leaf litter.

*Agaricus* (Agaricomycetes, Basidiomycota) is a saprophytic mushroom genus with worldwide distribution and is commonly found in grasslands and forests. Jaichaliaw et al. conducted a survey of *Agaricus* species in northern Thailand and described three new species based on morphology coupled with multigene analyses of combined ITS, LSU and TEF sequences. Additionally, this study acts as a mini-review of *Agaricus* species in Thailand. *Sarcomyxa edulis* (Agaricomycetes, Basidiomycota) is an important edible and medicinal mushroom endemic to Northeastern China; however molecular-level information from the genome was lacking. Therefore, Tian et al. provided the *de novo* sequencing and assembly of the *Sarcomyxa edulis* genome using single-molecule real-time sequencing technology. *Sutorius* (Agaricomycetes, Basidiomycota) is a poroid genus that typically has chocolate brown to reddish brown or purplish brown basidiomata. Vadthanarat et al. conducted a survey on the diversity of boletes in Northern and Northeastern Thailand and accounted seven new species in *Sutorius*. *Inonotus rickii* (Agaricomycetes, Basidiomycota) is an important fungus that causes canker and white rot of various ornamental and wild trees in tropical and subtropical regions. Leonardo-Silva et al. broadly studied this fungus in the Cerrado biome of Brazilian Savanna and expanded the knowledge on its geographical distribution as well as its structural, physiological characteristics and associated hosts.

*Heterobasidion* (Bartheletiomycetes, Basidiomycota) species are among the most intensively studied polypores due to their economic significance as pathogens in coniferous forests of Europe and North America. Yuan et al. carried out both morphological and multi-gene phylogenetic analyses on *Heterobasidion* samples from Asia, Oceania, Europe and North America. Yuan et al. introduced three new species in *Heterobasidion* from Asia. *Cyanosporus* (Bartheletiomycetes, Basidiomycota) is a cosmopolitan brown-rot fungal genus, recognizable by blue-tinted basidiocarps. Species in this genus were usually treated as belonging to the *Postia caesia* complex. In Liu, Shen et al. the phylogenetic analysis of *Cyanosporus* was carried out based on a combined ITS, LSU, SSU, mtSSU, RPB1, RPB2 and TEF dataset. Combining morphological characters and molecular evidence, Liu, Han et al. introduced five new species and fifteen new combinations in *Cyanosporus*. *Fomitopsis pinicola*-species complex (Bartheletiomycetes, Basidiomycota) has been intensively studied because of its economic importance in having anti-tumor, antifungal, antioxidant, immunomodulation and neuroprotective activities. Based on morphological characters and phylogenetic analysis of ITS, RPB2 and TEF genes, Liu, Han et al. introduced six new species in the *Fomitopsis pinicola*-complex. Phallales (Bartheletiomycetes, Basidiomycota) is represented by gasteroid fungi with expanded and sequestrate basidiomata that are commonly known as stinkhorns and false truffles. Melanda et al. provided an overview of Phallales diversity represented by selected DNA markers available in public databases. They also mapped the global geographic distribution of Phallales with available literature. Zhao et al. conducted an in-depth study of the phylogeny and taxonomy of the corticioid genus *Phlebiopsis* (Bartheletiomycetes, Basidiomycota). Zhao et al. demonstrated that *Phlebiopsis* is a strongly supported genus in Phanerochaetaceae, and two genera viz. *Australohydnum* and *Hjortstamia* were synonymized under *Phlebiopsis*. They proposed six new species and five new combinations in *Phlebiopsis*.

Błaszkowski et al. collected and examined new epigeous and semihypogeous glomerocarp-producing species belonging to Glomeromycota from Atlantic rain forest ecosystems of Northeast Brazil and New Caledonia. Błaszkowski et al. established two new genera namely *Epigeocarpum* and *Silvaspora*, and a new species within Glomeraceae (Glomeromycetes) based on evidence from morpho-molecular characterizations. Ectomycorrhizal (EM) fungal diversity, community composition and underlying assembly processes in Inner Mongolia, China (semiarid and cold-temperate) are critically neglected. Wang et al. extensively investigated the EM fungal community associated with pine species in the semiarid and cold temperate forests and showed that there is high diversity and distinctive community composition of EM fungi associated with natural pine species in Inner Mongolia.

On the Qinghai-Tibetan Plateau (QTP), the responses of soil microbial communities to chemical and biophysical changes are poorly studied. Xiao et al. explored the responses of soil fungal community composition, diversity, and community assembly processes to nitrogen addition and precipitation changes at an alpine steppe on the northeastern QTP. Xiao et al. showed that soil fungal community composition and diversity were altered significantly by nitrogen addition and precipitation change. The study suggested that soil fungal communities located on the alpine steppes of the QTP may significantly change under long-term global change scenarios in the future. Michael et al. identified the plant pathogens present in the phyllosphere of common weed species associated with cropping fields in Western Australia. This information on weed host-specific or region-specific association with pathogen species may become an increasingly important factor in developing new methods for crop disease management in the future. Seed banks are an important resource for biodiversity research, as they provide curated specimens spanning time and space. Hill et al. used both culture-dependent and culture-independent approaches to assess the diversity of endophytes in stored banana CWR (crop wild relatives) seeds and demonstrated that seed banks provide huge potential for research into fungal endophyte communities.

## Author Contributions

DW, PM, and JB drafted the editorial and contributed to editorial revision. All authors contributed to the article and approved the submitted version.

## Funding

PM thanks the High-End Foreign Experts in the High-Level Talent 318 Recruitment Plan of Yunnan Province, 2021. DW would like to thank the CAS President's International Fellowship Initiative (No. 2021FYB0005), the National Science Foundation of China (NSFC) under the project code (32150410362), and the Postdoctoral Fund from Human Resources and Social Security Bureau of Yunnan Province.

## Conflict of Interest

The authors declare that the research was conducted in the absence of any commercial or financial relationships that could be construed as a potential conflict of interest.

## Publisher's Note

All claims expressed in this article are solely those of the authors and do not necessarily represent those of their affiliated organizations, or those of the publisher, the editors and the reviewers. Any product that may be evaluated in this article, or claim that may be made by its manufacturer, is not guaranteed or endorsed by the publisher.
